# Malignant Pheochromocytomas/Paragangliomas and Ectopic Hormonal Secretion: A Case Series and Review of the Literature

**DOI:** 10.3390/cancers11050724

**Published:** 2019-05-24

**Authors:** Anna Angelousi, Melpomeni Peppa, Alexandra Chrisoulidou, Krystallenia Alexandraki, Annabel Berthon, Fabio Rueda Faucz, Eva Kassi, Gregory Kaltsas

**Affiliations:** 1 Department of Internal Medicine, Unit of Endocrinology, National and Kapodistrian University of Athens, Laiko hospital, Goudi, 11527 Athens, Greece; evakassis@gmail.com; 2 Endocrine Unit, 2nd Department of Internal Medicine Propaedeutic, Research Institute and Diabetes Center, National and Kapodistrian University of Athens, Attikon University Hospital, 12462 Haidari, Greece; moly6592@yahoo.com; 3 Unit of Endocrinology, Theagenio Cancer Hospital, 2 Al Simeonidi Str., 54007 Thessaloniki, Greece; a.chrisoulidou@gmail.com; 4 1st Department of Propaedeutic Internal Medicine, National and Kapodistrian University of Athens, Laiko hospital, Goudi, 11527 Athens, Greece; alexandrakik@gmail.com (K.A.); gkaltsas@endo.gr (G.K.); 5 Section on Endocrinology and Genetics, Eunice Kennedy Shriver National Institute of Child Health and Human Development, National Institutes of Health, Bethesda, MD 20892, USA; annabel.berthon@nih.gov (A.B.); fabio.faucz@pucpr.br (F.R.F.); 6 Department of Biological Chemistry, Medical School, National and Kapodistrian University of Athens, Goudi, 11527 Athens, Greece

**Keywords:** metastatic OR malignant pheochromocytoma, paraganglioma, ectopic secretion, lL-6, normetanephrines

## Abstract

Malignant pheochromocytomas (PCs) and paragangliomas (PGLs) are rare neuroendocrine neoplasms defined by the presence of distant metastases. There is currently a relatively paucity of data regarding the natural history of PCs/PGLs and the optimal approach to their treatment. We retrospectively analyzed the clinical, biochemical, imaging, genetic and histopathological characteristics of fourteen patients with metastatic PCs/PGLs diagnosed over 15 years, along with their response to treatment. Patients were followed-up for a median of six years (range: 1–14 years). Six patients had synchronous metastases and the remaining developed metastases after a median of four years (range 2–10 years). Genetic analysis of seven patients revealed that three harbored succinate dehydrogenase subunit B/D gene (SDHB/D) mutations. Hormonal hypersecretion occurred in 70% of patients; normetanephrine, either alone or with other concomitant hormones, was the most frequent secretory component. Patients were administered multiple first and subsequent treatments including surgery (n = 12), chemotherapy (n = 7), radionuclide therapy (n = 2) and radiopeptides (n = 5). Seven patients had stable disease, four had progressive disease and three died. Ectopic hormonal secretion is rare and commonly encountered in benign PCs. Ectopic secretion of interleukin-6 in one of our patients, prompted a literature review of ectopic hormonal secretion, particularly from metastatic PCs/PGLs. Only four cases of metastatic PC/PGLs with confirmed ectopic secretion of hormones or peptides have been described so far.

## 1. Introduction

Malignant pheochromocytomas (PCs) and paragangliomas (PGLs) are rare neuroendocrine tumors with an incidence of less than 1/1,000,000, defined by the presence of metastatic disease in non-chromaffin tissues without considering recurrent or locally invasive tumors [1,2,3]. The WHO Endocrine Tumor Classification 4th Edition (2017) [4] prompted the new concept that all PGLs have some metastatic potential and assigned an International Classification of Diseases for Oncology (ICD-O)-3 (malignant tumors) for all PGLs, surpassing the previous categories of benign and malignant tumors in favor of an approach based on risk stratification [4]. 

Long-term follow-up has shown that PCs/PGLs exhibit a 15–20% 10-year probability of recurrence and up to 20% malignancy rate [5]. In the presence of metastatic disease, a wide 5-year survival rate range, from 40 to 77%, has been described, as well as a heterogeneous progression free survival (PFS) ranging from 4 to 36 months following various therapeutic modalities [6,7,8]. Synchronous metastases at initial diagnosis are encountered in 10% in PCs and 34% in PGLs but can occur up to 20 years after initial diagnosis with the most common metastatic sites being regional lymph nodes, bone (50%), liver (50%) and lung (30%) [9,10].

Although surgical removal is the mainstay of treatment of PCs/PGLs, further risk stratification regarding their malignant potential is required to define the follow-up protocols after complete resection [9]. A number of histopathological scores have been developed to denote the malignant potential of these neoplasms such as the Adrenal Pheochromocytoma and Paraganglioma (GAPP) Score used to evaluate the malignant potential of both PCs and sympathetic PGLs, and the Pheochromocytoma of the Adrenal Gland Scaled Score (PASS) used to evaluate the malignant potential of PCs only, although it exhibits a relatively low predictive value (sensitivity 50% and specificity 45%) [11,12]. Besides these scores, a number of clinical characteristics and biomarkers have also been proposed to predict the metastatic potential of PCs/PGLs including younger age at presentation [5,13,14], larger sized (>5 cm) tumors [5,15], extra-adrenal location of the neoplasm [5], and higher circulating norepinephrine levels [5,15,16]. However, the majority of these markers exhibit a relatively low positive predictive value. Currently the presence of inactivating mutations of the succinate dehydrogenase subunit B (*SDHB*) gene is strongly associated with the development of metastatic PCs and PGLs [17].

The natural history of patients with malignant PCs/PGLs is divergent as approximately half of the patients with metastatic PCs/PGLs have stable disease (SD) one year after diagnosis without any therapeutic intervention [18,19]. Although the timing of further therapeutic interventions for metastatic PCs/PGLs has not been clearly defined, further therapeutic options for symptomatic patients in the presence of progressive disease include cytoreductive surgery, systemic chemotherapy (using either the combination of cyclophosphamide, vincristine, and dacarbazine (CVD) or temozolomide) and/or ^131^I- metaiodobenzylguanidine (^131^I-MIBG) [20]. Recently, some data have also emerged for the activity of peptide receptor radionuclide therapies (PRRTs) that bind to somatostatin receptors expressed by such neoplasms [19]. Percutaneous ablation has also been used as a minimally invasive local treatment option [20].

Ectopic secretion of bioactive compounds (hormones or peptides) from PCs or PGLs is rare; approximately 1.3% of all PCs may produce ectopic adrenocorticotropic hormone (ACTH) secretion; in rare cases, ectopically secreted corticotropin releasing hormone (CRH) may also occur [21]. Case reports have also described the production of parathyroid hormone related-peptide (PTHrP), vasoactive intestinal peptide (VIP), vasopressin, growth hormone releasing hormone (GHRH), insulin, somatostatin, aldosterone, renin, interleukin-6 (IL-6), and neuropeptide Y. However, the majority of ectopic secretion is encountered in non-metastatic PCs/PGLs, whereas there is paucity of data regarding its prevalence in metastatic PCs/PGLs.

In this study, we retrospectively analyzed the clinical, biochemical, radiological and genetic features of patients with metastatic PCs/PGs, along with the therapeutic modalities employed and their response to various treatments. These data were compared with the currently existing data on metastatic PCs/PGLs, focusing on the ectopic secretion of biologically active compounds from metastatic PCs/PGLs. 

## 2. Results

### 2.1. Epidemiological and Clinicopathological Data

Fourteen patients with a median age of 45 years (interquartile range (IQR): 30) were included in the present study; seven patients with metastatic PCs, six with metastatic PGLs and one with both PC and PGL. The median follow-up period was six years (range: 1–14, IQR: 10 years) (Table 1). Half of the patients (50%) presented with synchronous metastases (5 PGLs, 2 PCs); 69% of all cases developed metastases in the distal lymph nodes (cervical and abdominal); 46% in the liver, 23% in the bones and 15% in the lung. In one patient with a bladder PGL, metastases were found in the aortopulmonary window and the heart (substantiated by dedicated cardiac magnetic resonance imaging (MRI)). The remainder of the patients developed metastases after a median time of four years from diagnosis (range 0.8–10 years, IQR = 8) in the liver, lung, distal lymph nodes and vertebrae. 

Ten patients (71%) had functional tumors; three had normetanephrine hypersecretion, four had concomitant normetanephrine and metanephrine secretion, one had only dopamine secretion, one had normetanephrine and dopamine secretion, and one had normetanephrine, metanephrine and dopamine secretion. All patients presented with relevant symptoms attributed to hormonal hypersecretion, or symptoms due to compression of nearby tissues, except two cases in whom the diagnosis was made incidentally (Τable 1).

In nine patients, the diagnosis of malignant PC/PGL and radiological follow-up was performed with MRI, which showed increased signal intensity in the T2-weighted sequence, whereas in the remaining five patients, initial diagnosis as well as radiological follow-up were performed with computed tomography (CT). ^131^I-MIBG was performed and found to be positive in eight out of ten patients. Octreoscan was performed in seven patients showing increased uptake in the primary tumor and metastases in four, ^68^Gallium-Labeled (1,4,7,10-tetraazacyclododecane-*N*,*N*’,*N*’’,*N*’’’-tetraacetic acid)-1-NaI3-octreotide(^68^Ga-DOTANOC) was performed in three patients and showed increased uptake in all, whereas ^18^F-fluorodeoxyglucose positron emission tomography (^18^F-FDG PET) was positive in nine out of eleven patients (Table 1).

The median size of the neoplasms was 4.25 cm (IQR: 4). Histological confirmation was performed in 12 patients and all patients with PCs had a PASS > 6 (Table 2). All tumors were positive for chromogranin and synaptophysin immunostaining. The mean Ki-67% proliferative indices of the primary and metastatic sites were 11 ± 3.8% and 44 ± 7%, respectively. Three tumors out of five showed intense immunochemical staining for somatostatin receptor 2, with two of them also staining for somatostatin receptor 5 (Table 2).

Out of seven patients tested for germline mutations, two had *SDHB* mutations and one had a *SDHD* mutation (Table 1). One patient with a *SDHB* mutation had a functional left PC, secreting normetanephrine and dopamine. It was treated initially by surgery but developed metastases in the vertebrae after six years. The second was a female patient with concomitant presence of PC and multiple abdominal PGLs that were non-secretory. The patient with the *SDHD* mutation was a female with a functional bladder PGL secreting dopamine and multiple PGLs in the cervical spine, the aorto-pulmonary window and the carotid.

### 2.2. Treatment and Outcome

**Figure 1:**
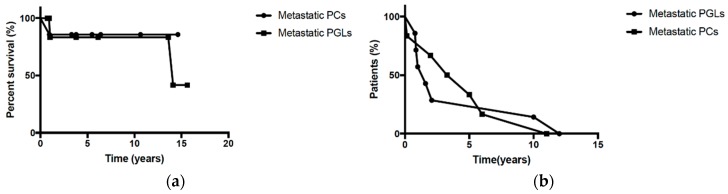
(**a**). Overall survival (OS) of malignant pheochromocytomas (PCs) and paragangliomas (PGL) (median OS for PGLs = 14 years, IQR: 11.7) (**b**) Median progression free survival (PFS) until the presence of the first or new metastases: malignant PCs: 4.14 years (IQR: 3.38) and PGLs: 1.6 years (IQR: 1.03) (p = 0.8). Abbreviations: MPCs: metastatic pheochromocytoma, MPGLs: metastatic paragangliomas, IQR: interquartile range.

As first line treatment, nine patients underwent radical surgical resection of the primary tumor (all R0), four patients were treated with chemotherapy (cisplatin/etoposide or capecitabin/temozolomide) and one with ^131^I-MIBG. Three patients exhibited SD until the last follow-up and no further treatment was needed: the first was treated with ^131^I-MIBG and the two others with surgical resection of the primary and metastases. One patient who was treated with chemotherapy died from the disease and the remaining ten developed progressive disease (PD) and received second line treatment. Two patients were treated with chemotherapy (cisplatin/etoposide), one of whom died, three with radionuclides (^131^I-MIBG (n = 1), ^177^Lu-DOTATE (n = 2)), three with repeated surgical resection and two with local radiotherapy. Four patients developed SD after second line treatment (two had been treated with PRRT, the third with surgical debulking and the fourth with radiotherapy), whereas the remaining five developed PD; two had no further treatment and were followed-up. Three patients received third line treatment; two with temozolomide (one died after three cycles) and the third with chemotherapy (cisplatin/etoposide) and subsequently with ^131^I-MIBG (4th line treatment). Overall, at the end of the follow-up period, seven patients exhibited SD (50%), three (2 PGLs, 1PCs) died (21%) and four developed PD (29%). Patients with PCs developed PD after a median time of 4.14 years (IQR: 3.38) following initial treatment, whereas patients with PGL developed PD after a median time of 1.6 years (IQR: 1.03) (p = 0.8). Median overall survival (OS) for PGLs was 14 years (IQR: 11.7) (Figure 1a,b).

### 2.3. Ectopic Secretion and Review of the Literature

**Figure 2:**
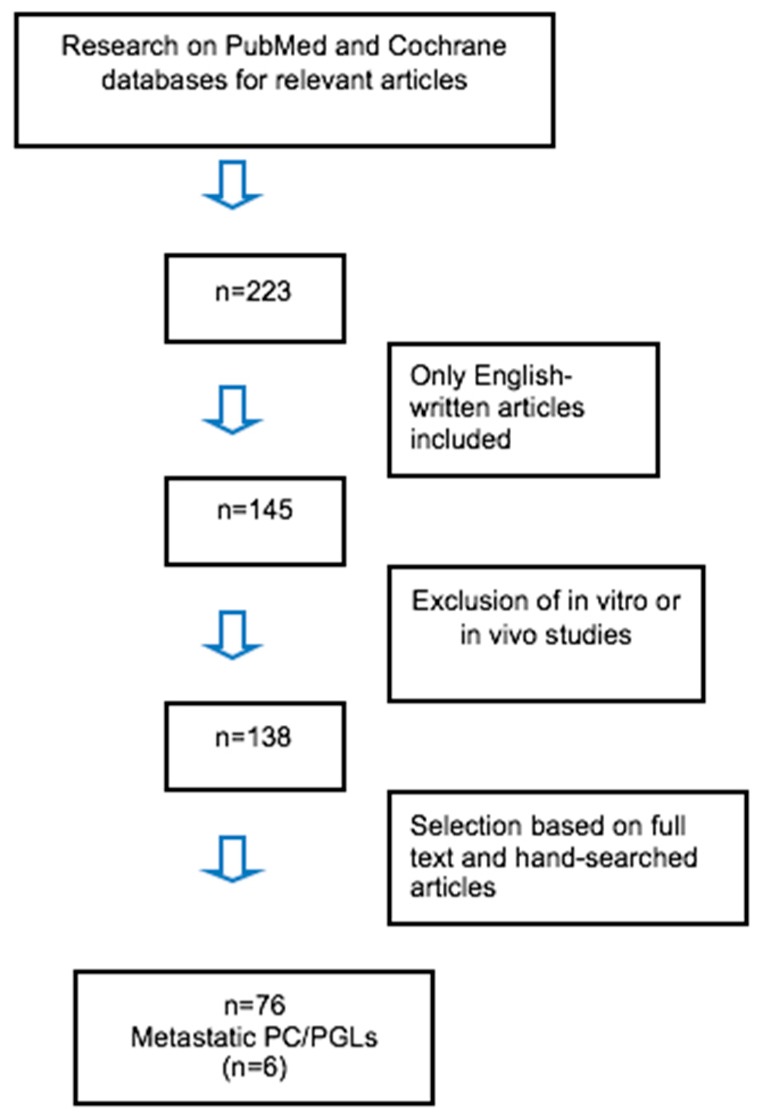
Flow diagram.

One patient with non-functional PGLs and synchronous metastases to the vertebrae and muscles developed pyrexia not attributed to an infectious state and that was resistant to anti-inflammatory drugs. IL-6 levels were measured and were found to be elevated at 236 pg/mL (normal values < 7). Besides blood analysis, immunohistochemical staining confirmed the higher cytoplasmic expression of IL-6 in the paraffin-embedded tissue of the patient’s PGL compared to PC tissue of a patient without fever (control tissue), which showed weaker staining. Following surgical debulking, the pyrexia improved but recurred due to PD and the patient was treated with chemotherapy (cisplatin and etoposide) and temozolomide, but died one year after the initial diagnosis. A systematic review of the literature revealed seventy-six relevant English language articles, mainly case reports, addressing ectopic secretion of bioactive compounds of PC/PGLs over the last 30 years (Figure 2).

A total of 150 cases (Table 3) with ectopic secretion from PCs/PGLs have been reported (data presented in Table 3). ACTH secretion accounted for 33% of all cases (n = 49), whereas CRH, VIP, vasopressin, PTH, renin, aldosterone, insulin or somatostatin, GHRH, and neuropeptide Y secretion have also been described [16,21,22,23,24,25,26,27,28,29,30,31,32,33,34,35,36,37,38,39,40,41,42,43,44,45,46,47,48,49,50,51,52,53,54,55,56,57,58,59,60,61,62,63,64,65,66,67,68,69,70,71,72,73,74,75,76,77,78,79,80,81,82,83,84,85,86,87,88,89,90,91,92,93,94,95,96,97,98]. IL-6 secretion has already been reported in 40 cases with PCs/PGLs (Table 3). Only four case reports with confirmed ectopic secretion from metastatic PCs/PGLs have been described (data presented in Table 4). In particular, regarding metastatic PCs, one secreted ACTH and another one secreted PTHrP. For metastatic PGLs, one secreted ACTH and another secreted ACTH and IL-6. There was one case of PC with suspicion of IL-6 secretion without laboratory confirmation and a PGL with suspicion of IL-b and tumor necrosis factor (TNF) secretion without biochemical confirmation. In all cases, patients with IL-6 ectopic secretion presented with pyrexia resistant to any treatment, which resolved after surgical debulking of the tumor (Table 4).

## 3. Discussion

In the present study we present our experience from a series of 14 malignant PCs/PGLs treated with multiple therapeutic modalities. Three patients (2 PGLs, 1PC) died as result of the disease after a median follow-up of six years. Despite multiple therapeutic modalities, seven out of eleven patients (63%) exhibited PD; patients with PGLs appear to have more rapid PD (1.16 years) compared to patients with PCs (4 years), although due to the relatively small number of patients included, this was not statistically significant. One of the patients with a metastatic PGL presented with refractory pyrexia due to ectopic secretion of IL-6, confirmed by elevated IL-6 levels in the serum and histological confirmation of IL-6 protein expression in the tissue. Systematic review of the literature showed that although ectopic secretion of IL-6 is relatively common in benign PCs/PGLs, only one further case of metastatic PGL with confirmed IL-6 and ACTH secretion has been reported [75].

In a recent large study including 330 PCs/PGLs, the incidence of metastatic PCs/PGLs was 6.9% [99]. The risk of metastases was associated with an age at diagnosis ≤35 years (hazard ratio [HR] 2.74, [95% Confidence Interval (CI) 1.19–6.35), tumor size ≥6.0 cm (HR 2.43, 95% CI 1.06–5.56), extra-adrenal location (HR 2.73, 95% CI 1.10–7.40), and tumor producing only normetamephrine (HR 2.96, 95% CI (1.30–6.76)) [100]. In our series the median age of 45 years was higher, yet similar, to the mean age (41 ± 17 years old) of the metastatic group of the previous study. The median size of the primary tumor was smaller (4.25 cm), whereas the hormonal profile was similar, showing that secretion of norepinephrine was the predominant secretory component.

Epinephrine-secreting tumors, either alone or with norepinephrine, originate exclusively from the adrenal gland [10]. Norepinephrine-secreting PGLs are tumors in which norepinephrine only or norepinephrine plus dopamine are produced; 50% of PCs and 100% of PGLs are of this type [10]. In our series 64% of the patients with malignant PCs/PGLs (3 with PGLs, 5 with PCs, 1 with a PC and PGL) had either norepinephrine secreting PCs/PGLS or norepinephrine in combination with epinephrine or dopamine. Previous studies are in line with these data, reporting that the metastatic ratio is twice as high in norepinephrine-secreting PCs compared to epinephrine-secreting PCs [10]. Norepinephrine-secreting PGLs lack phenylethanolamine N-methyltransferase, the enzyme that converts ormetanephrine to metanephrine and are considered less differentiated than adrenaline-producing (metanephrine) tumors [10]. In addition, dopamine hypersecretion is considered a feature of immaturity and a marker for metastatic PGLs [15]. Dopamine-secreting PGLs are typically non-symptomatic; it has been reported that the plasma level of methoxytyramine, the O-methylated metabolite of dopamine, is 4.7-fold higher in patients with metastases than in those without, suggesting its use as a potential biomarker [100].

Only half of our patients showed uptake in octreoscan, whereas 82% exhibited increased uptake in ^18^F-FDG-PET and 80% in ^131^I-MIBG. Data in the literature have shown that ^131^I-MIBG and ^18^F-FDG-PET exhibit higher sensitivity than octreoscan [101]. However, it subsequently became apparent that the sensitivity of ^68^Ga- 1,4,7,10-tetraazacyclododecane-1,4,7,10-tetraacetic acid (DOTA) tyrosine-3-octreotate (DOTATATE)-PET/CT imaging in patients with PCs/PGLs seems to be higher than that of ^131^I-MIBG scintigraphy and ^18^F-FDG-PET in mapping metastatic PCs/PGLs [101,102,103,104]. In our series, three cases out of five (one PC and two PGLs) showed immunochemical expression of somatostatin receptor 2, and in two of them this was concomitant with receptor 5.

Currently, there are no systemic therapies approved by the European Medicines Agency or the US Food and Drug Administration (FDA) for patients with metastatic PCs/PGLs [17,18]. Surgical resection or debulking is the gold standard of treatment. Other treatment options for non-operable tumors are limited to chemotherapy (CVD) with relatively low response rates (complete response in 4%, partial response in 37% and SD in 14%) and inappropriately high toxicity [105,106,107]. Nevertheless, chemotherapy is considered part of the initial management in patients with metastatic *SDHB*-related PGLs (median of 20.5 cycles) [107]. Lately there is increased interest in the use of PRRT in malignant PCs/PGLs [108,109]. Recent studies including patients with metastatic PCs/PGLs treated with ^90^Y-DOTATATE or ^177^Lu-DOTATATE have shown a mean progression free survival (PFS) (36% had PD and 50% SD) and OS of 39 and 61 months, respectively, compared to conventional ^131^I-MIBG treatment (mean PFS: 14 months and OS: 23 months) [110,111]. In our series, three patients with metastatic PCs/PGLs were treated with ^131^I-MIBG and two with ^177^Lu-DOTATATE either as first, second, third or fourth line treatment; two of them developed PD and three SD during the last follow-up, whereas the median PFS was 3.5 years (range: 0.05–11.8, IQR = 7.3), which is the longest compared to the other therapeutic modalities employed. These data appear encouraging although larger series are required.

One of our patients with a non-functional malignant PGL presented with pyrexia resistant to any treatment due to IL-6 ectopic secretion. Ectopic secretion of hormones or peptides, from PCs/PGLs is rare (approximately 1% of all PCs), encountered mostly in non-malignant and non-genetic cases. It is probable that ectopic secretion of bioactive compounds from these tumors is often overloooked or clinical manifestations are masked by the hypersecretion of catecholamines. The most frequent ectopically-secreted hormone is ACTH, mostly reported in benign PCs [21,22,23,24,25,26,27,28,29,30,31,32,33,34,35,36,37,38,39,40,41,42,43,44,45,46,47,48,49,50,51]. Ectopic secretion of hormones or peptides from malignant PCs/PGLs has been reported even more scarcely. In particular, only four cases of metastatic PCs/PGLs with biochemically or immunohistochemically confirmed ectopic secretion of hormones or peptides have been described in the English literature so far [25,30,75,80]. In two other cases of malignant PCs/PGLs, ectopic secretion has been suspected but not confirmed biologically or immunohistochemically [97,98].

IL-6 ectopic secretion has already been reported in 40 cases of PCs/PGLs, however only one of them, a case of cervical PGL, was metastatic [64,65,66,67,68,69,70,71,72,73,74,75,76,77]. The reason for the high level of IL-6 expression in PCs/PGLs is unclear. It has been suggested that IL-6 over-production can be either ascribed directly to the tumor or indirectly accounted for by tumoral production of the high circulating norepinephrine levels [74]. However, the presence of IHC expression of IL-6 protein in the PGL tissue of our patient with ectopic IL-6 secretion is more in favor of IL-6 synthesis and secretion by the PGL neoplastic cells.

## 4. Methods

### 4.1. Patients

In this retrospective study, data were obtained from three Greek Endocrine Units; the Endocrine Unit of Laiko Hospital (n = 11) and the Endocrine Unit of Attiko Hospital (n = 2) of the National and Kapodistrian University of Athens and the Theagenio Hospital in Thessaloniki (n = 1). The medical records of patients with metastatic PCs/PGLs over a period of 20 years (1998–2018) were reviewed by two independent researchers (Anna Angelousi and Krystallenia Alexandraki) in order to collect the clinico-pathological characteristics of these patients along with imaging and biochemical findings. In addition, the therapeutic response to the various utilized treatments was also recorded. 

The study protocol was approved by the Ethics or Audit Committees of all participating centers. All patients gave informed consent according to the Declaration of Helsinki and Good Clinical Practice guidelines. Informed consent was obtained from patients’ relatives in the case of death. The ethical code number is AP 450 and the date of decision of approval from the ethical committee of the “General Laiko” hospital of Athens is 8 April 2019.

Patients with the following criteria were included in our study: (i) histopathological and/or biochemical and/or imaging confirmation of the diagnosis of primary PCs/PGLs and distant metastases; (ii) available data during the follow-up period. Exclusion criteria included: (i) benign PCs/PGLs; (ii) patients with documented venous or loco-regional or proximal lymph node spread only. For the systematic review of the literature, ectopic secretion was defined as ectopic production, involving the synthesis and secretion of bioactive compounds (peptides or hormones) from benign or malignant tumors that do not normally synthesize and secrete these particular compounds.

### 4.2. Review of the Literature on Ectopic Hormonal Secretion

To identify studies and determine their eligibility, a systematic review was conducted in the PubMed and Cochrane Databases. Search terms included the following: “pheochromocytoma OR metastatic pheochromocytoma”, “paraganglioma OR metastatic paraganglioma”, “paraneoplastic syndrome”, “ectopic secretion”, “IL-6”. The above keywords were also combined with the Boolean operators AND and OR. Two of the authors (Anna Angelousi and Eva Kassi) independently examined all potentially eligible titles and abstracts. Full manuscripts were obtained as necessary to finalize eligibility. Reference lists of eligibility studies were also searched through to identify additional studies. Only English language papers were selected. Studies with hormonal ectopic secretion from tumors other than PCs/PGLs were also excluded as well as in vitro studies. Seventy-six articles were finally included (Figure 2).

### 4.3. Hormonal Secretion

All patients had 24 h urinary metanephrine and normetanephrine levels measured by high-performance liquid chromatography. IL-6 levels were measured with High–Performance Liquid Chromatography (HPLC) (Bio-Rad, Athens, Greece).

### 4.4. Imaging

All patients underwent conventional imaging with either CT or MRI along with functional imaging including ^131^I-MIBG, ^111^In-pentetreotide (Octreoscan) and ^68^Gallium labelled octreotide, or ^18^F-fluorodeoxyglucose positron emission tomography (^18^F-FDG PET). In all patients primary tumor and metastases were detected and followed-up with conventional imaging (five patients with CT and nine with MRI). In addition, all patients had at least one form of functional imaging. 

### 4.5. Statistical Analyses

All statistical analyses were conducted using GraphPad Prism Version 6 for Mac OS X (GraphPad Software, La Jolla, California, USA). Quantitative values are reported as median (interquartile range (IQR) and/or 25–75% range) or mean ± standard deviation (SD), and categorical variables as percentages. Overall survival (OS) was defined by time from diagnosis of PCs/PGLs to death by any cause. Progression free survival (PFS) was defined from the time of the initiation of a specific treatment to presence of new metastases or progression of the existing ones (according to Response Evaluation Criteria in Solid Tumors (RECIST)). OS and PFS were estimated using Kaplan–Meier curves. Comparison of the PFS and OS between patients with metastatic PCs and patients with metastatic PGLs was performed using the Wilcoxon test (GraphPad Software, La Jolla, California, USA) A p value < 0.05 was considered significant.

## 5. Conclusions

Malignant PCs/PGLs are a rare entity that can metastasize many years after surgical resection of the primary tumor, even 10 years after the initial diagnosis as in our case. In our series metastatic PGLs appear to have more rapid PD (1.16 years) compared to patients with PCs (4 years). Available treatments are, so far, non-curative; further research is needed to evaluate therapies with novel mechanisms of action. PRRT seems to improve the outcome of our patients with metastatic PCs/PGLs, resulting in longer PFS, but should be studied in larger clinical trials. Ectopic secretion of a number of bioactive compounds from PCs/PGLs is rare and becomes extremely rare in malignant ones according to the literature. However, it could be overlooked and should always be considered, especially when patients present with unusual symptoms that cannot be totally attributed to catecholamine hypersecretion. 

## Figures and Tables

**Table 1:** Epidemiological and clinicopathological characteristics of the studied population.

Characteristics	
**Number (PC)**	14 (7)
**Female sex, n (%)**	8 (57%)
**Median age (IQR), years**	45 (30)
**Size primary tumor (cm)**	4.25 (4)
**Sychronous/ Metachronous metastases**	7/7
**Functionality n, (%)**	10 (71%)
-normetanephrines	3
-metanephrine	0
-normetanephrines and metanephrines	4
-dopamine	1
-normetanephrine and dopamine	1
-normetanephrines. metanephrnes, dopamine	1
**Functional imaging**	
-Octreoscan (positive, %)	4/7 (57%)
-^68^Gallium labelled octreotide (positive, %)	3/3 (100%)
-^18^F-FGD-PET (positive, %)	9/11 (82%)
-^131^I-MIBG (positive, %)	8/10 (80%)
**Follow-up, median (IQR, range), years**	6 (10, 1–14)
**Treatment (any line)**	
-Surgery	12 (86%)
-PRTTs (^131^I-MIBG or ^17^Lu-Dotate)	5 (35%)
-Chemotherapy	7 (50%)
-Radiotherapy	2 (14%)
**Genetic status (n)**	7
-SDHB+	2/7
-SDHD+	1/7
**Mortality**	3 (21%)

Abbreviations: PC: pheochromocytoma, IQR: interquartile range, PRRTs: peptide receptor radionuclide therapy, SDHB/D: succinate dehydrogenase subunit B/D.

**Table 2:** Tumors’ (PCs and PGLs) characteristics.

Characteristics	N (%)
**Primary tumor location, n (%)**	
*-PC*	7 (50%)
*-PGL*	6 (46%)
bladder PGL	1
para-aortic PGL	2
paravertebral PGL	1
abdominal PGL	2
*-PCs + PGLs*	1
**Primary tumor size, mm (median, IQR)**	4.25, 4
**Location of metastases**	
-lymph nodes (abdominal/cervical)	9 (69%)
-liver	6 (46%)
-lung	2 (15%)
-bones	3 (23%)
**Metastases per patient**	>2 (1–4)
**Histopathological data**	
-Ki-67 (mean ± SD)	11 ± 3.8%
-PASS	7.75
-SSTR2,5(positive/total (n))	(3/5)

Abbreviations: PC: pheochromocytoma, PGL: paraganglioma, SD: standard variation, PASS: Pheochromocytoma of the Adrenal Gland Scaled Score, SSTR 2,5: somatostatin receptor (2, 5).

**Table 3:** Ectopically secreted bioactive compounds from PCs/PGLs based on the literature.

Hormone	No of Cases	PCs	PGLs	Malignant (n)	References
Total	150	137	13	5	
-ACTH	49	43	6	2 (1PC, 1PGLs)	[21,22,23,24,25,26,27,28,29,30,31,32,33,34,35,36,37,38,39,40,41,42,43,44,45,46,47,48,49,50,51]
-CRH	8	6	2	0	[52,53,54,55,56,57,58,59]
-VIP	6	6	0	0	[33,60,61,62]
-Vasopressin	1	1	0	0	[57]
-Calcium	1	1	0	0	[63]
-IL-6	40	39	1	1	[64,65,66,67,68,69,70,71,72,73,74,75,76,77]
-PTH/PTHrp	17	17	0	1	[78,79,80,81,82]
-Calcitonin	5	5	0	0	[33,83,84,85]
-GH/GHRH	7	6	1	0	[86,87,88,89,90]
-Insuline/IGF-1	1	0	1	0	[91]
-Somatostatin	1	1	0	0	[92]
-Aldosterone	1	1	0	0	[33]
-Renin	2	2	0	0	[33,93]
-CRH and ACTH	2	1	1	0	[59,94]
-CRH or ACTH and vasopressin	2	2	0	0	[57,58]
-IL b	1	1	0		[95]
-ACTH and IL-6	1	0	1	1	[76]
-Calcitonin and VIP	3	3	0	0	[33,63,83]
-PTH and aldosterone	1	1	0	0	[96]
-Neuropeptide Y	1	1	0	0	[16]

Abbreviations: PCs: pheochromocytomas, PGLs: paragangliomas, ACTH: adrenocorticotropic hormone, CRH: corticotropin-releasing hormone, VIP: vasoactive intestinal peptide, IL-6: interleukin-6, PTH: parathormone, PTHrp: parathyroid hormone related-peptide, GH: growth hormone, GHRH: growth hormone releasing hormone, IGF-1:insulin growth factor-1. IL-b: interleukin b.

**Table 4:** Ectopic secretion of bioactive compounds from malignant PCs/PGLs based on the literature.

References	Number	PCs/PGLs	Metastases	Ectopic Secretion	Treatment
Kakudo K, et al. 1984 [25]	Case report (n = 1)	PC	Liver, lungs, bones, lymph nodes	ACTH (blood and tissue)	Surgery
Teno et al. 1996 [97]	Case report (n = 1)	PC	Bones	Suspicion of IL-6 but not measured	External Radiation
Tutal E et al. 2017 [30]	Case report (n = 1)	Renal PGL	Lymph nodes	ACTH (blood and tissues)	Surgery
Omura M et al. 1994 [75]	Case report (n = 1)	Cervical PGL	Bones	ACTH, IL-6 (blood and tumor)	Surgery and chemotherapy
Mutabagani KH et al. 1999 [98]	Case report (n = 1)	Mediastinal PGL	Liver	Anemia (probably Il-1 and anti-TNF secretion but never measured)	Surgery and hepatic arterial chemoembolization
Bridgewater JA et al. 1993 [80]	Case report (n = 1)	PC	Left para-aortic lymph node	PTHrp (blood and tissue)	Surgical resection

Abbreviations: PCs: pheochromocytomas, PGLs: paragangliomas, ACTH: adrenocorticotropic hormone, IL-6: interleukin-6, PTHrp: parathyroid hormone related-peptide, anti-TNF A: anti-tumor necrosis factor A.
